# Impact of Climate Change and Heat Stress on Milk Production in Korean Holstein Cows: A Large-Scale Data Analysis

**DOI:** 10.3390/ani13182946

**Published:** 2023-09-17

**Authors:** Eunjeong Jeon, Seungho Jang, Joon-Mo Yeo, Dong-Wook Kim, Kwanghyun Cho

**Affiliations:** 1 Dairy Science Division, National Institute of Animal Science, Rural Development Administration, Cheonan 31000, Republic of Korea; jeonej12@korea.kr; 2 Department of Animal Science, Jeonbuk National University, Jeonju 54896, Republic of Korea; gh5576@naver.com; 3 Department of Dairy Science, Korea National University of Agriculture and Fisheries, Jeonju 54874, Republic of Korea; yeoj@korea.kr; 4 Department of Poultry Science, Korea National University of Agriculture and Fisheries, Jeonju 54874, Republic of Korea; poultry98@korea.kr

**Keywords:** big data, climate change, heat stress, Holstein cows, milk production

## Abstract

**Simple Summary:**

Climate change driven by global warming and greenhouse gas emissions is a major global concern, particularly in Republic of Korea. Heat stress is a prominent issue in livestock farming, particularly in Holstein dairy cows, due to its negative impact on productivity. However, a limited number of studies have evaluated its effects in Republic of Korea. This study aimed to evaluate climate change trends in Republic of Korea and assess the impact of heat stress on milk production in Holstein cows using comprehensive large-scale data. The results showed that both milk production and lactation persistency sharply declined after surpassing their respective temperature-humidity index (THI) break points. Understanding the relationship between climate change, heat stress, and dairy cow productivity allows stakeholders to make informed decisions to ensure the sustainability and resilience of the livestock industry amid changing environmental conditions.

**Abstract:**

This study investigated the effects of heat stress on milk production in Korean Holstein cows using large-scale data. Heat stress was assessed using the temperature-humidity index (THI). Weather records (2016 to 2020) were collected from 70 regional weather stations using an installed automated surface observing system (ASOS). A dataset of 2,094,436 milk production records from 215,276 Holstein cows obtained from the Dairy Cattle Genetic Improvement Center was analyzed. Stepwise selection was used to select the input variables, including the daily maximum THI (THI_max). Least-squares means were calculated for milk yield, fat and protein corrected milk (FPCM), fat and protein yield, fat-to-protein ratio, solids not fat, and lactation persistency. Segmented linear regression analysis determined the break points (BPs) of the THI_max. Over the five years, heat stress exposure increased, particularly from May to September. This study identified BPs around THI_max of 80–82 for milk yield and FPCM. Similar patterns for other milk traits were observed, which significantly decreased beyond their respective BPs. These findings indicate that THI variations adversely affect milk yield and composition in dairy cows, highlighting the importance of appropriate feeding management strategies to ensure the optimal productivity of Holstein cows under varying climatic conditions.

## 1. Introduction

Climate change is a global concern, driven by increased global warming and greenhouse gas emissions. Climate change in Korea is also accelerating, with the average temperature in Korea rising by more than twice the global average temperature increase of 0.75 °C over the past 100 years (1911 to 2010), reaching 1.8 °C [1]. Furthermore, it is projected that by 2050, the average temperature in Korea will increase by 3.4 °C, accompanied by an increase in the number of heat wave days and tropical night days (Representative Concentration Pathway 8.5) [2].

Hot climate conditions significantly impact the agricultural sector, and the issue of heat stress in livestock farming is becoming increasingly prominent [3,4,5]. In the US livestock industry, it is estimated that economic losses resulting from heat stress reach billions of dollars yearly, with dairy cattle being the most affected group [6,7]. These economic losses arise because most animals are raised in environments where temperatures fall outside their comfort range [8]. The productivity of dairy cows is maximized when they are free from stress, can engage in normal physiological activities, and are sensitive to changes in environmental conditions, such as ambient temperature, humidity, wind, and sunlight [5,9]. Stress is defined as a discomforting physiological response that occurs when an individual is unable to cope with problems [10]. These can be broadly classified into three categories: stress caused by infection, chronic life stress, and acute sustained stress due to extreme cold or heat exposure [11]. Approximately 99% of dairy cows raised in Republic of Korea belong to the Holstein breed [12], which is susceptible to high temperatures and experiences heat stress when the ambient temperature exceeds the threshold of 27 °C [5,13,14]. Exposure of dairy cows to heat stress leads to decreased feed intake, milk yield, milk quality, and fertility rates [3,4,5], resulting in increased production costs and reduced income. Therefore, evaluating changes in dairy cow productivity due to heat stress, considering various environmental effects, should be regarded as a proactive strategy for establishing a stable production foundation in the livestock industry.

One evident benefit of utilizing data from public weather stations is their reliability, as these stations do not depend on individual measurements of body temperature or respiration, which can be challenging to gather on a large scale [3]. Previous studies proposed a methodology that employs a temperature-humidity index (THI) for evaluating animal heat tolerance by combining test-day records and public weather station data [3,15,16]. Ravagnolo and Misztal [17] also proposed the application of public weather data for national assessments. The THI is a numerical value that quantifies the degree of heat stress by using temperature and relative humidity [16,18]. Numerous studies have been conducted to estimate heat stress in dairy cows using this index [19,20,21]. Heat stress in cows begins to occur when the THI value exceeds 72, and it was reported that mild stress occurs at THI 72–79, moderate stress at THI 80–89, and severe stress at THI 90–98 [22,23,24]. Bohmanova et al. [19] reported that the THI threshold for stress in cattle varies depending on environmental factors and regional characteristics. Despite the ongoing warming trend due to climate change in Republic of Korea, there is limited research on the impact of heat stress on dairy cow productivity using large-scale data. Therefore, this study aimed to investigate the effect of heat stress on the milk productivity in Korean Holstein cows using large-scale data.

## 2. Materials and Methods

### 2.1. Data Collection and Preprocessing

#### 2.1.1. Climate Data and THI Calculation

Given the lack of farm-specific weather monitoring equipment on most Korean dairy farms, acquiring farm-level weather records is challenging. Instead, we utilized daily meteorological data from the weather stations closest to the farm locations. These records cover the period from July 2016 to December 2020. In total, weather records were collected from 70 regional weather stations equipped with an automated surface observing system (ASOS). The THI was used to estimate the degree of heat stress and was calculated using the following equation [25]:

THI_max = (1.8 × T_max_ +32) − [(0.55 − 0.0055 × RH) × (1.8 × T_max_ − 26.8)] (1)

where T_max_ and RH denote the daily maximum temperature (°C) and the relative humidity (%), respectively. We chose THI_max because it aligned most effectively with the information derived from public weather stations [17]. This choice was also driven by the fact that the test-day record of milk yield is more sensitive to the extreme values of the maximum THI than the daily average THI [26].

#### 2.1.2. Test-Day Daily Milk Production Records

Test-day records of milk yield, fat and protein corrected milk (FPCM), milk fat and protein yield, milk fat to protein ratio, solids not fat (SNF), and lactation persistency were collected from the Dairy Cattle Genetic Improvement Center (NongHyup Agribusiness Group Inc., Goyang-si, Republic of Korea) in Republic of Korea. Milk compositions were analyzed for milk fat, milk protein, and SNF using a Milkoscan (CombiFoss FT + 500 S/H, Hillerød, Denmark), which is a widely recognized and validated instrument for accurately measuring these parameters. The data covered a period of five years (2016–2020) and comprised a total of 6,011,160 data points. Only data from cows with monthly test records of at least ten tests per year were included in the analysis, and outliers were removed. Data points exceeding 400 days in milk (DIM) were excluded from the analysis. Ultimately, a dataset of 215,276 Holstein cows (122,087 primiparous cows and 93,189 multiparous cows with 2–5 parities) was used, encompassing 2419 farms and 2,094,436 records. Table 1 presents the descriptive statistics for the variables used in this study. The FPCM and milk fat to protein ratio [27] were calculated as:

FPCM (kg/d) = 0.327 × milk yield (kg) + 12.95 × milk fat (kg) + 7.2 × milk protein (kg)(2)

Milk fat to protein ratio = milk fat (%)/milk protein (%)(3)

Lactation persistence (%) can be represented by the simple formula [milk (kg) earlier test − milk (kg) later test] × 100, assuming a precise testing interval of 30 d. However, because of the practical difficulty in aligning the testing intervals of all cows to exactly 30 d, a calculation formula that considers this is needed. The formula employed in Republic of Korea to calculate lactation persistency (%) in Holstein dairy cows is as follows [28].

(4) $$\mathrm{Lactation}\;\mathrm{persistency}\;(\%)=\;\left[1-\frac{\left(\begin{array}{c} \mathrm{milk}\;\left(kg\right)\;earlier\;test\;-\\ milk\;\left(kg\right)\;later\;test \end{array}\right)\times \frac{30\;days}{days\;between\;tests}}{mlik\left(kg\right)\;earlier\;test}\right]\times 100$$

### 2.2. Input Variable Selection

The selection of optimum input variables is essential for when determining a suitable model structure [29]. This approach employs a stepwise regression procedure to identify the most significant predictors among the available input variables.

### 2.3. Modeling

We merged the test-day daily milk production records with the daily THI_max calculated from climate data. The following statistical model was utilized to estimate the impact of heat stress on milk traits. The statistical significance of differences between the least-squares averages of the groups was assessed by applying the following null hypothesis at a significance level of 1%:

H_0_ : LSM(i) = LSM(j) (5)

where LSM (i(j)) refers to the least square mean (LSM) estimate of the i(j)_th_ effect (i ≠ j).

Y_ijklmn_ = H_i_ + Y_j_ + P_k_ + THI_max_l_ + S_m_ + DIM_n_ + e_ijklmn_ (6)

where Y_ijklmn_ is an observation of test-day records for milk traits (milk yield, FPCM, milk fat and protein yields, milk fat to protein ratio, SNF, and lactation persistency), H_i_ is the fixed effect of the region (i = 1–13), Y_j_ is the fixed effect of test year (j = 1–5), P_k_ is the fixed effect of parity (k = 1–5), THI_max_l_ is the temperature-humidity index as expressed by THI_max (l = 40–95), S_m_ is the fixed effect of test season (m = 1–4; spring: March to May, summer: June to August, autumn: September to November, winter: December to February), DIM_n_ denotes the days in milk (n = 1–400), and e_ijklmn_ represents the vector of random residual effects. Values are presented as LSM and standard errors of the mean unless otherwise stated. The LSM of milk traits in different THI was used as the dependent variable. The segmented linear regression model assumes the following slope:

𝑦𝑖^∗^ = a + b_1_X_i_ + e_i_; when X_i_ ≤ BP 𝑦𝑖^∗^ = a + b_1_X_i_ + b_2_(X_i_ − BP) + e_i_; when X_i_ > BP (7)

LSM of milk traits in different THI_max was denoted as 𝑦𝑖^∗^, with an intercept (a) and regression coefficients (b_1_ and b_2_). The regression coefficient b_1_ was applied when THI_max X_i_ was lower than the break point (BP), while b_2_ was applied when THI_max X_i_ exceeded the BP. The random residual term was represented as e_i_. The BP was defined as the appropriate threshold value of THI_max. Linear regression was employed to analyze the effect of heat stress.

### 2.4. Statistical Analysis

All statistical analyses were performed using the R software [30]. Descriptive statistics were calculated using the psych package and visualized using the ggplot2 package. The mass package was also used to select the input variables through a stepwise analysis. The emmeans package was used to estimate the LSM and standard errors of the milk production traits in the models. Considering the linear significance of heat stress, the BP of the THI was evaluated using segmented regression analysis using the R Segmented package.

## 3. Results and Discussion

### 3.1. Climate Change Trends in Republic of Korea

**Figure 1:**
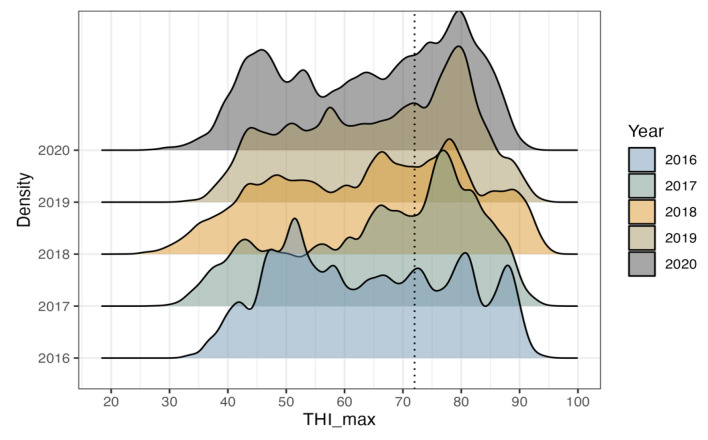
Plot illustrating the distribution of annual THI_max data from 2016 to 2020 in Republic of Korea. Each year is represented by a different color, and the dotted line within the figure indicates the THI_max threshold of 72. THI, temperature-humidity index; THI_max, maximum temperature-humidity index.

The distribution of the annual THI_max data from 2016 to 2020 in Republic of Korea was examined using the weather data employed in this study (Figure 1). The frequency of data points with THI values exceeding 72 has increased over the years. In dairy cows, heat stress is known to occur when the THI exceeds 72 [22,23,24], and it has been reported that the climate of Republic of Korea is transitioning from temperate to subtropical [31]. Previous studies have indicated that the average temperature in Republic of Korea has surged at a rate more than double the global average temperature increase of 0.75 °C over the past century, recording a rise of 1.8 °C [1]. Furthermore, predictions suggest that by 2050, the average temperature in Korea could escalate by as much as 3.4 °C. Consequently, it has been suggested that the changing climate in Republic of Korea is gradually becoming detrimental to Holstein cow productivity.

**Figure 2:**
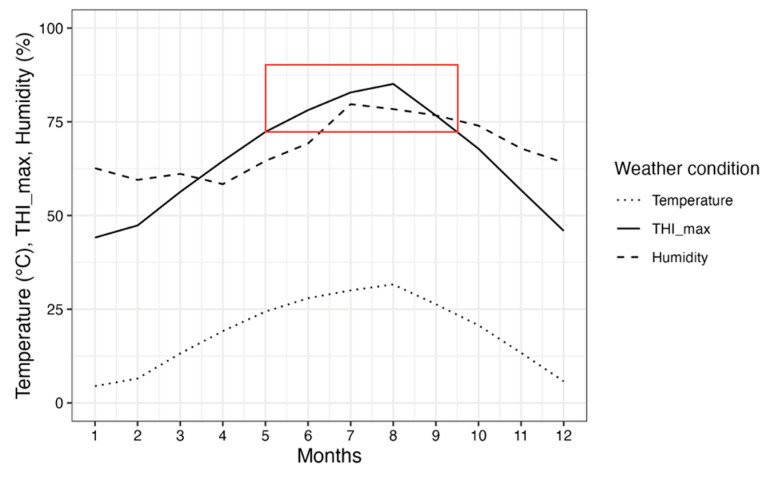
The monthly average temperature, THI_max, and humidity during 2016–2020 in Republic of Korea. A different line style represents each weather condition, and the red box corresponds to points where THI_max exceeds 72. THI_max, maximum temperature-humidity index.

The monthly average temperatures, THI_max, and relative humidity are shown in Figure 2. This figure presents a combination of data collected over a period of five years, revealing consistent patterns in weather factors, where temperature and humidity increase, and THI_max also increases. Figure 2 shows the notable occurrence of heat stress from May to September, as evidenced by the THI_max values surpassing the critical threshold of 72. Consequently, during this period, animals in Republic of Korea experienced repeated exposure to heat stress.

In the summer, animals exposed to high environmental temperatures and humidity struggle to regulate their body temperature unless conditions fall within a comfortable range known as the thermoneutral zone [19]. Previous research suggests that these animals can dissipate the heat they have accumulated during the day, but only if the nighttime air temperature drops below 21 °C [7]. Bohmanova et al. [19] stated that lowering the THI below 72 is not feasible even by applying evaporative cooling techniques from June to August. This explains the notable decline in milk production observed from June to August. Additionally, the authors emphasized a significant point that milk production showed signs of recovery after heat stress, with improvements becoming evident in October. This timing aligns with THI levels dropping below the threshold of 72, which indicates an interplay between THI and milk production dynamics during heat stress.

Atmospheric temperature and humidity in Republic of Korea vary greatly depending on the season and THI [32,33]. Unlike other regions, Republic of Korea is bordered by water, leading to higher temperatures and humidity levels during the summer. Given this context, Republic of Korea’s environmental conditions differ from those of other countries, underscoring the importance of Republic of Korea’s specific temperature and humidity factors in studying heat stress in Holstein cows.

### 3.2. Impact of Heat Stress on Milk Yield and Compositions

**Figure 3:**
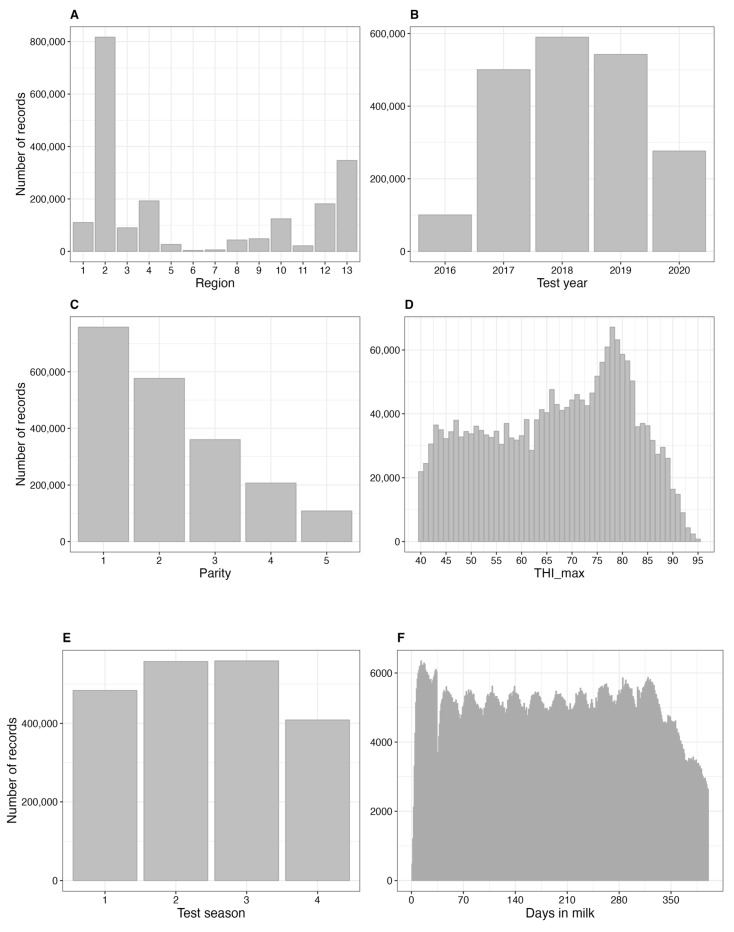
Bar plots depicting the data distribution of input variables in the model. Input variables represent (**A**) region, (**B**) test year, (**C**) parity, (**D**) THI_max (maximum temperature-humidity index), (**E**) test season, and (**F**) days in milk.

The following variables were selected using the stepwise regression model: region, test year, parity, THI_max, test season, and DIM (*p* < 0.001). The data distributions of the input variables used in the model are shown in Figure 3.

**Figure 4:**
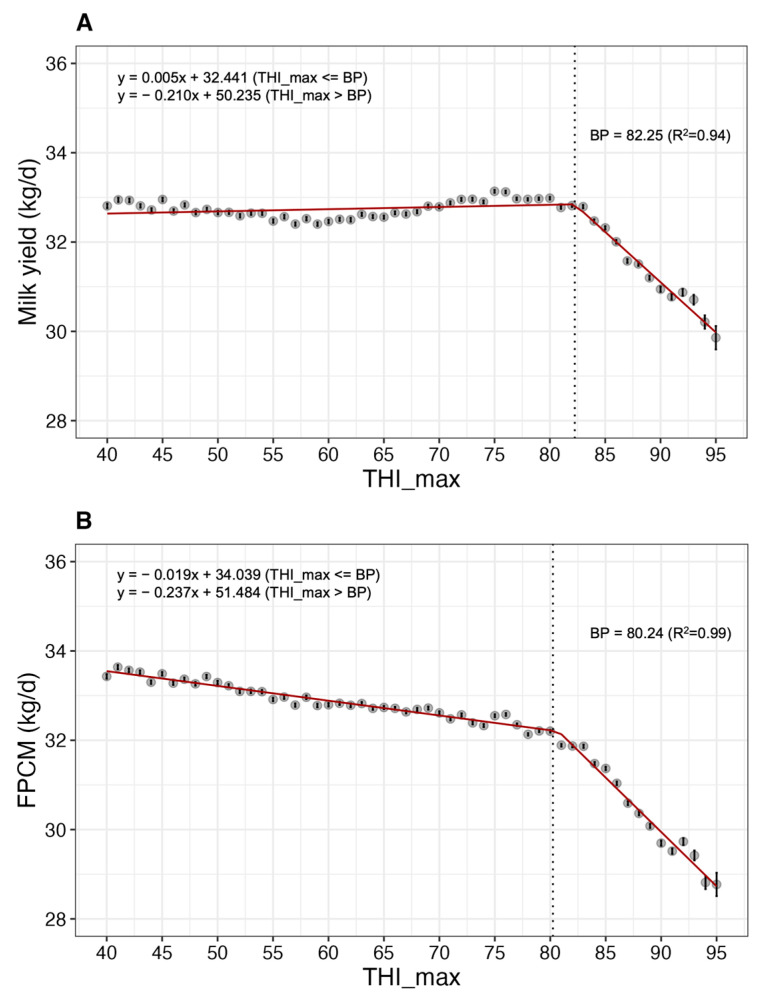
Least-square mean (LSM; solid circles) and standard error (bars) indicate the association between heat stress (measured by THI_max) and (**A**) milk yield and (**B**) fat and protein corrected milk (FPCM) in Korean Holstein cows (*p* < 0.05). THI_max, maximum temperature-humidity index; BP, break point.

**Figure 5:**
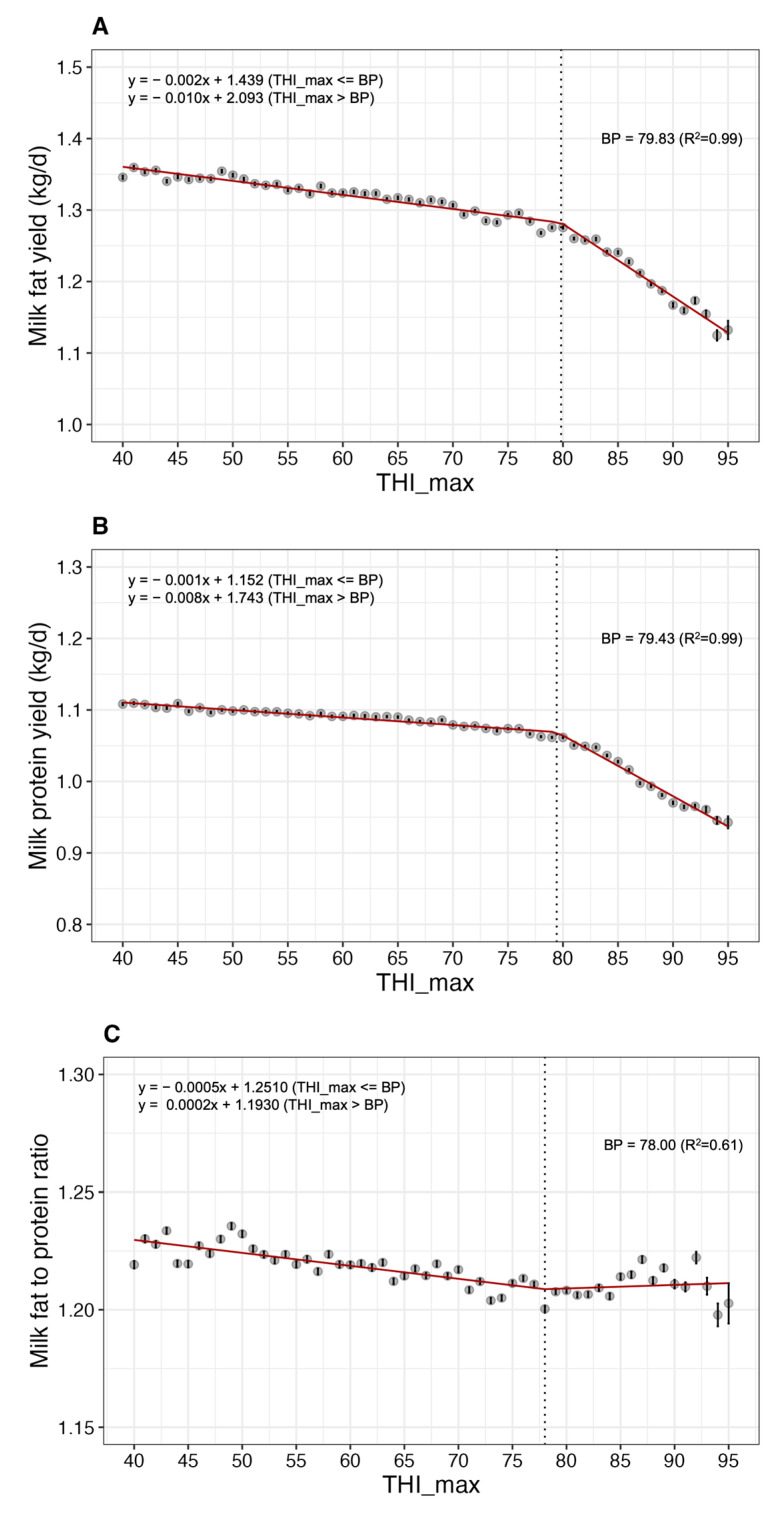
Least-square mean (LSM; solid circles) and standard error (bars) indicate the association between heat stress (measured by THI_max) and (**A**) milk fat yield, (**B**) milk protein yield, and (**C**) milk fat to protein ratio in Korean Holstein cows (*p* < 0.05). THI_max, maximum temperature-humidity index; BP, break point.

**Figure 6:**
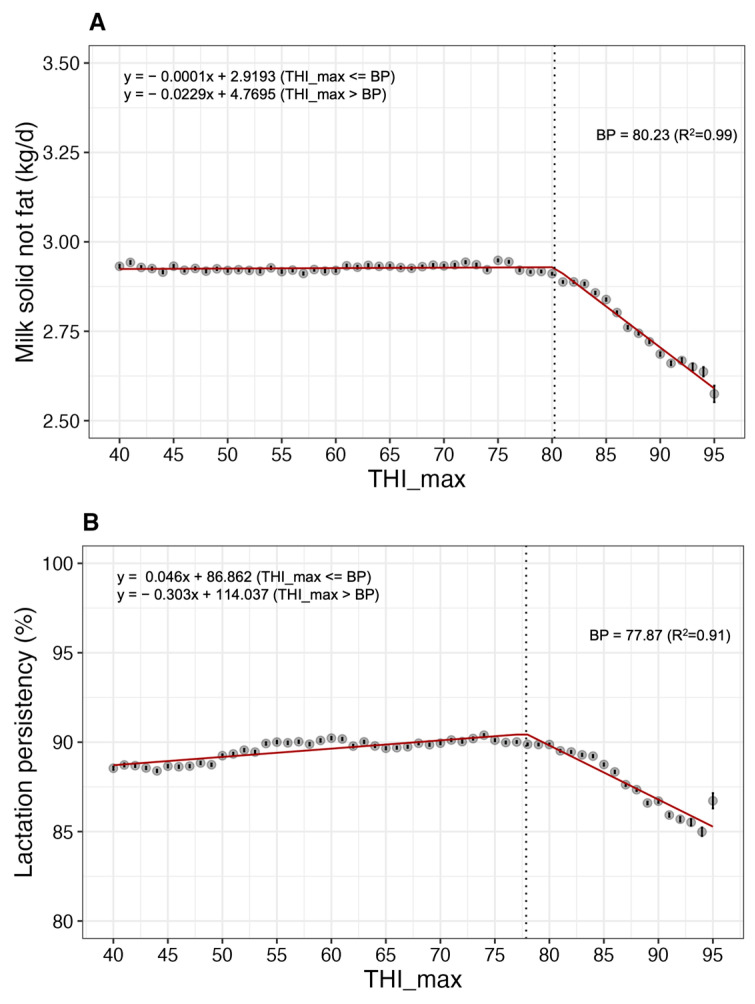
Least-square mean (LSM; solid circles) and standard error (bars) indicate the association between heat stress (measured by THI_max) and (**A**) milk solids not fat (SNF) and (**B**) lactation persistency in Korean Holstein cows (*p* < 0.05). THI_max, maximum temperature-humidity index; BP, break point.

Considering these diverse environmental factors, we calculated the LSM of milk yield, FPCM, fat and protein yields, milk fat to protein ratio, SNF, and lactation persistency in relation to the variations in THI_max (Figure 4, Figure 5 and Figure 6). The findings indicated that the BP for milk yield with respect to THI was 82.25 (Figure 4A). As THI increased from 82 to 95, there was a decline in milk yield from approximately 32.82 kg to 29.86 kg. Therefore, when THI exceeded a BP of 82.25 and increased to 95, a milk yield reduction of approximately 9.02% was observed. The BP for FPCM in relation to THI was found to be 80.24 (Figure 4B). Beyond this point, an increase in THI was associated with a decrease of approximately 10.54% in FPCM.

The BPs for milk fat and protein yields were identified as 79.83 and 79.43, respectively (Figure 5A,B). Similarly, as the THI increased from these respective BPs to 95, milk fat and protein yields decreased by approximately 11.23% and 11.18%, respectively. The BP for milk fat to protein ratio was determined to be 78.00, exhibiting deviations in the LSM distribution compared to that of other milk yield and milk component parameters (Figure 5C).

The BP for SNF was 80.23, and beyond this threshold, a decrease of approximately 11.55% in SNF was observed (Figure 6A). The lactation persistency tended to decrease when it exceeded BP of 77.87 (Figure 6B).

It is generally recognized that dairy cows experience heat stress when the THI exceeds 72, and various studies have provided threshold values and corresponding signs of heat stress for dairy cows [22,23,24,33]. When THI levels ranged from 72 to 79, the cows experienced mild stress. In this range, cows adapt to the climate by seeking shade, increasing their respiration rates, and expanding their blood vessels. However, its effects on milk production within this range have been reported to be minimal. When the THI ranges from 80 to 89, cows are subjected to moderate stress, reducing milk productivity and reproductive efficiency. Cows in this range have been reported to exhibit increased saliva production, respiration rate, water consumption, body temperature, and decreased feed intake. Studies have reported reduced milk protein, milk fat, and SNF levels due to heat stress [33,34,35]. Bohmanova et al. [19] suggested that the decrease in milk components such as milk protein and milk fat, due to heat stress could be attributed to the dilution effect caused by decreased feed intake and increased water consumption. Our results also showed a decrease in milk yield, FPCM, and milk components (milk fat, milk protein, and SNF) when the THI exceeded 80, consistent with findings of earlier studies. In contrast, Hammami et al. [4] reported decreased milk production when THI reached 62. Rensis et al. [36] observed that cows displayed mild signs of heat stress when the experimental conditions were set to have a THI exceeding 68. The variation in the critical THI for milk production could be attributed to various environmental factors, such as the climatic region, presence of cooling systems, and the level of genetic improvement, all of which influence the relationship between THI and milk production. When THI levels reach 90–98, dairy cows experience severe heat stress, resulting in a sharp decline in milk productivity and reproductive efficiency [33]. Additionally, cows can undergo distress caused by increased body temperature, accelerated respiration (panting), and excessive saliva secretion, resulting in potential health complications. Finally, THI levels exceeding 98 could cause mortality in cows. Heat stress can increase cortisol levels and affect milk production in cows [6,37]. Bohmanova et al. [19] reported that seasonal differences in milk production are caused by periodic environmental changes throughout the year, which directly affect milk production through reduced dry matter intake and indirectly through fluctuations in the quantity and quality of feed. Lim et al. [38] demonstrated that the increasing rate of decline in the milk yield of cows in Republic of Korea could be explained by higher heat production. Furthermore, high-production cows are more vulnerable to heat loads [39]. If the effects of heat stress persist, a reduction in milk yield can be observed even after the heat load period has subsided, and milk production may not revert to the initial levels following exposure [40].

These findings demonstrate the negative impact of THI variation on milk yield and composition in dairy cows, highlighting the importance of implementing appropriate feeding management strategies that consider climatic factors to ensure the productivity of Holstein cows. Therefore, mitigating heat stress in dairy cows is imperative when the THI exceeds a specific BP. It can be achieved by implementing a more comprehensive approach to feeding management and installing an effective farm cooling infrastructure. Previous studies have indicated that common strategies for mitigating the impact of heat stress include providing adequate shade and ensuring proper facilities, such as utilizing evaporative cooling systems [41,42]. Providing shade is considered an economical and readily accessible approach to alleviate the adverse effects of heat stress in animals, as it prevents direct exposure to solar radiation [13]. Another approach involves considering nutritional aspects, including dietary factors, such as dietary fiber, dietary fat, microbial additives, vitamins, and other anti-stress additives [8]. Dietary fiber and fat in the feed are readily available and recognized as nutrients that can alleviate the adverse effects of heat stress in animals [43].

One limitation of this study was utilizing climate data at the regional level rather than farm-specific meteorological data, as there is a lack of dairy farms in Republic of Korea equipped with on-farm weather monitoring devices. Therefore, this study used weather data from the closest locations to calculate the THI. Although this approach may not capture the individual environments of each farm, it allowed us to reflect the general climatic conditions experienced by the cows within the corresponding regions. Using regional weather station data, we sought to mitigate potential inaccuracies and inconsistencies due to reliance on farm-level data obtained using less advanced instrumentation. For a more accurate and reliable assessment of the impact of heat stress on milk productivity, further research using farm-specific meteorological data is required.

## 4. Conclusions

This study provides valuable insights into the effects of heat stress on milk production in Korean Holstein cows using large-scale data. The analysis revealed an increase in the frequency of THI values exceeding 72 over the five-years, indicating a growing prevalence of heat stress in dairy cows. This study identified critical BPs for THI at which milk yield, FPCM, milk components, such as fat and protein yield, and lactation persistency, experienced sharp declines. The BP for milk yield and FPCM were found to be 82.25 and 80.24, respectively. Additionally, BPs for milk fat and protein yields were identified at 79.83 and 79.43, respectively, while the BP for the milk fat to protein ratio was determined to be 78.00. Furthermore, the specific BP for SNF was determined to be 80.23, with a notable decrease in lactation persistency observed beyond a BP of 77.87. Beyond these respective BPs, THI variations also negatively affect milk productivity and composition in dairy cows. The observed reductions in milk yield and milk components were consistent with the findings from previous studies on heat stress in cows. These results emphasize the importance of implementing appropriate feeding management strategies that consider climatic factors to ensure the optimal productivity and resilience of Holstein cows under changing environmental conditions. Further research incorporating farm-specific meteorological data is warranted to provide a more accurate assessment of the impact of heat stress on milk production in dairy farms.

## Figures and Tables

**Table 1:** Descriptive statistics of the milk records (*n* = 2,094,436) for the 2016–2020.

Variables	Mean	CV (%) ^(3)^	SD ^(4)^	Median	Min	Max
Parity	2.17	0.55	1.19	2.00	1.00	5.00
Days in milk (d)	193.02	0.58	111.62	193.00	1.00	400.00
Milk yield (kg/d)	32.65	0.26	8.33	32.20	9.80	55.50
Fat and protein corrected milk (kg/d)	32.53	0.25	8.01	32.11	5.72	87.71
Milk fat yield (kg/d)	1.30	0.29	0.38	1.27	0.05	5.26
Milk protein yield (kg/d)	1.07	0.23	0.25	1.07	0.04	4.00
Milk fat to protein ratio ^(1)^	1.22	0.20	0.24	1.20	0.04	21.67
Milk solids not fat (kg/d)	2.90	0.25	0.72	2.88	0.47	21.19
Lactation persistency (%)	94.32	0.12	11.43	95.00	21.00	119.00
THI_max ^(2)^	65.56	0.23	15.07	67.40	21.61	96.70

^(1)^ Milk fat to protein ratio = milk fat (%)/milk protein (%). ^(2)^ THI_max = (1.8 × T_max_ + 32) − [(0.55 − 0.0055 × H) × (1.8 × T_max_ − 26.8)]. ^(3)^ CV (%) = coefficient of variation. ^(4)^ SD = standard deviation.

## Data Availability

The data are not publicly available due to General Data Protection Regulation.
